# Neurodermatitis of the scalp associated with trichotillomania treated with roflumilast cream 0.3%

**DOI:** 10.1016/j.jdcr.2024.12.004

**Published:** 2024-12-14

**Authors:** Edith Hanna, Nour El Moussawi

**Affiliations:** a Department of Dermatology, Centre Hospitalier Regional du Grand-Portage, CISSS du Bas-St-Laurent, Riviere-du-Loup, Quebec, Canada; b Faculty of Medicine, Universite Laval, Quebec City, Quebec, Canada; c Department of Dermatology, American University of Beirut Medical Center, Beirut, Lebanon

**Keywords:** neurodermatitis, PDE-4 inhibitor, PDE-4 treatment for neurodermatitis, phosphodiesterase-4 (PDE-4) inhibitor, roflumilast, roflumilast 0.3%, trichotillomania

## Introduction

Neurodermatitis of the scalp is a chronic skin condition that results in lichenification of the skin secondary to excessive scratching or excoriations.^1^^,^^2^ It affects mostly women over the age of 35.^1^^,^^2^ Treatment options include topical and intralesional steroids, topical capsaicin, or topical tacrolimus.^1^^,^^2^ Systemic treatments such as sedative antihistamines or antipsychotics have also been used.^1^^,^^3^ Roflumilast cream 0.3% is a selective phosphodiesterase-4 inhibitor approved by the Food and Drug Administration in 2022 for the treatment of psoriasis, and later in its foam form for the treatment of seborrheic dermatitis.^4^^,^^5^ We report the case of a 76-year-old female with neurodermatitis of the scalp associated with trichotillomania that was successfully treated with roflumilast cream 0.3%.

## Case report

**Fig 1:**
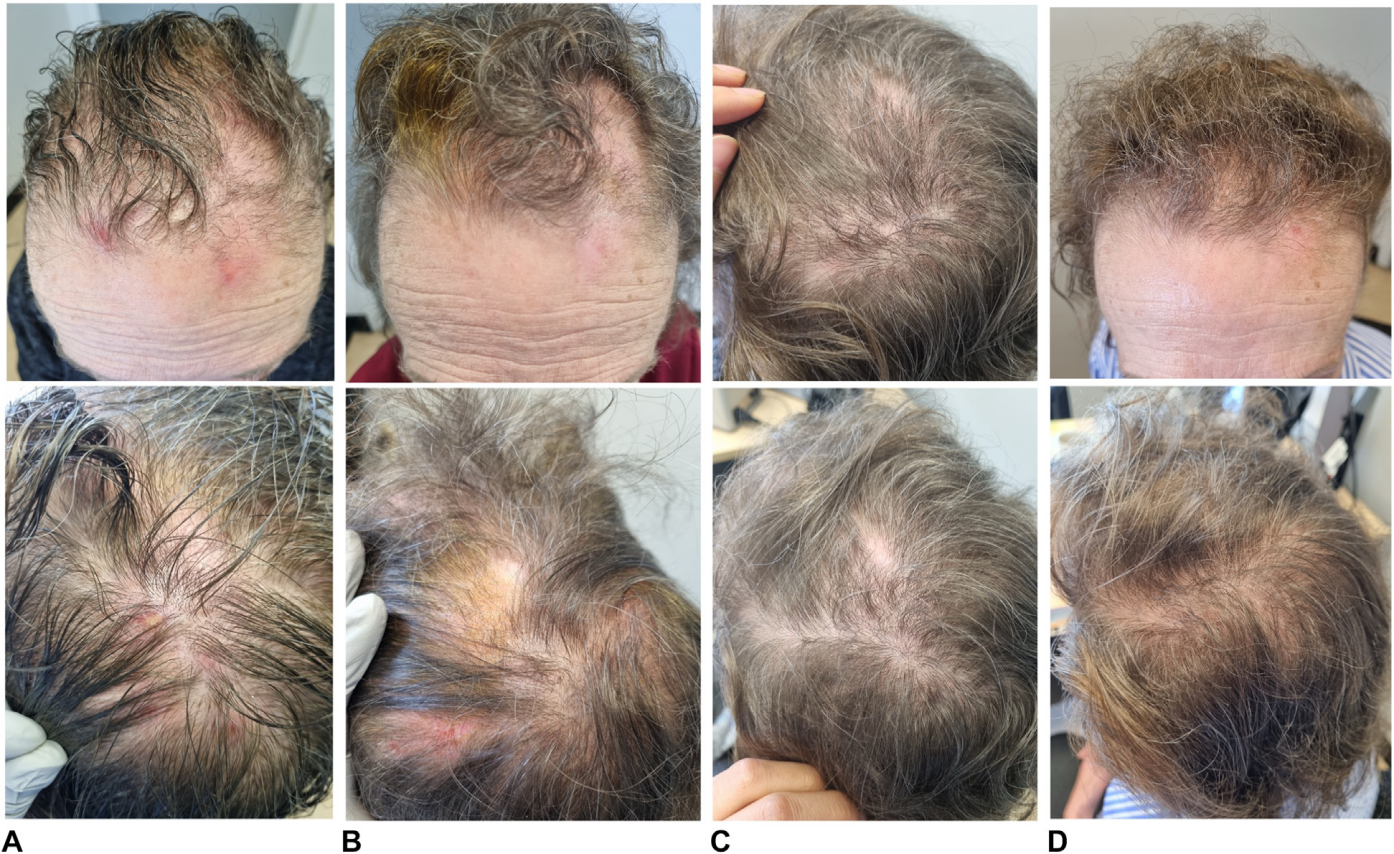
Before initiation of topical roflumilast cream 0.3% (**A**), after using it twice daily for 1 month (**B**), then after using it once daily for 2 months (**C**), and finally after 6 months of initiating treatment (**D**).

A 76-year-old female, known to have diabetes mellitus type II, presented to the dermatology outpatient department with a chronic, intense itch of the scalp of more than 8 years duration (Fig 1, *A*). Patient attributed her itching to insect bites and thus was seen by a psychiatrist who diagnosed her with delusions of parasitosis. She had failed multiple pharmacologic treatments including but not limited to citalopram, venlafaxine, clonazepam, pregabalin, sertraline, aripiprazole, and quetiapine. After consulting a dermatologist, she was diagnosed with neurodermatitis of the scalp in association with her delusions of parasitosis. She eventually failed a multitude of treatments such as calcipotriol/betamethasone gel, ketoconazole shampoo, terbinafine cream, intramuscular triamcinolone injection, N-acetylcysteine, cefadroxil, fusidic acid cream, mometasone cream, and betamethasone dipropionate lotion. On physical exam, multiple excoriated erythematous papules, nodules, and plaques were visualized, associated with alopecic patches demonstrating broken hairs of different lengths. The clinical diagnosis of a neurodermatitis associated with trichotillomania was made.

She was prescribed roflumilast cream 0.3% twice daily for 1 month (Fig 1, *B*), to be decreased to once daily for another month. Patient noted an 80% improvement with a decrease in itching and number of nodules on the scalp. Follow-up 2 months later showed postinflammatory erythema, only one persistent nodule on the scalp out of the 10 previously described nodules, and hair regrowth measuring around 1 mm (Fig 1, *C*). She was told to continue roflumilast cream 0.3% 3 times weekly for another 2 months. On follow-up 2 months later, patient denied any new lesions or itching. Physical exam showed complete hair regrowth with hairs measuring more than 3 mm in all 3 alopecic patches and disappearance of previously described erythematous excoriated papules and plaques. No scars were noted (Fig 1, *D*).

## Discussion

Neurodermatitis of the scalp is a chronic skin condition that can result in features of lichen simplex chronicus. This can manifest as thickened and hyperpigmented plaques marked by accentuated scaling and alopecia as a result of hair breakage.^1^^,^^2^ In this condition, the intense urge to scratch is a hallmark and is precipitated by pruritus secondary to either psychological stress, scalp dysesthesia, chronic medical conditions, such as diabetes mellitus, or pruritic skin diseases.^1^^,^^2^ The feeling of satisfaction that ensues after the act of scratching precipitates an itch-scratch cycle, which contributes to the persistence and progression of the lesions.^1^

Management is usually very challenging and should first be directed towards interrupting the itch-scratch cycle. This can be attempted by targeting the trigger, be it a psychological or a medical one, through proper psychological and medical evaluation. Treatment options aiming at decreasing the itching include topical or systemic steroids, antihistamines, topical tacrolimus, salicylic acid, capsaicin, gabapentin, and antipsychotic drugs, each with different efficacy and side effect profiles.^1^^,^^2^ Thus, other treatment options with similar effect and more tolerated side effect profile might be of benefit.

Studies have shown that phosphodiesterase-4, an enzyme expressed in keratinocytes and inflammatory cells, is upregulated in some inflammatory conditions.^6^ Normally, this enzyme catabolizes cyclic adenosine monophosphate, a signaling molecule for multiple pro-inflammatory cytokines.^6^ Thus, inhibiting phosphodiesterase-4 will cause an accumulation of cyclic adenosine monophosphate in inflammatory cells and, in turn, a decrease in the inflammatory profile dictated by the production of cytokines.^7^ Clinical trials were successful in proving the efficacy of roflumilast cream in reducing itch-related sleep loss and improving the quality of life of patients with psoriasis.^8^ The foam formulation of the drug showed similarly promising outcomes when it comes to itching in the context of seborrheic dermatitis.^9^

In our patient’s case, roflumilast cream 0.3% could represent an alternative topical treatment for neurodermatitis of the scalp with less pronounced side effects. This case report also highlights the importance of identifying other coexisting diseases that can help provide the best treatment option with a multidisciplinary approach tailored to the patient. Further research is warranted to better understand the interaction between this disease and other common diseases of the scalp as well as the benefits and risks of roflumilast cream or foam in its treatment.

## Conflicts of interest

None disclosed.

## References

[1] Lotti T., Buggiani G., Prignano F. (2008). Prurigo nodularis and lichen simplex chronicus. Dermatol Ther.

[2] Starace M., Iorizzo M., Mandel V.D. (2022). Scalp dysaesthesia and lichen simplex chronicus: diagnostic and therapeutic update with literature review. Clin Exp Dermatol.

[3] Ambika H., Vinod C.S., Sushmita J. (2013). A case of neurodermatitis circumscipta of scalp presenting as patchy alopecia. Int J Trichology.

[4] Arcutis Biotherapeutics Inc (2024). Zoryve (roflumilast) cream 0.3%. https://www.accessdata.fda.gov/drugsatfda_docs/label/2024/215985s007lbl.pdf.

[5] Arcutis Biotherapeutics Inc (2023). Zoryve (roflumilast) foam 0.3. https://www.accessdata.fda.gov/drugsatfda_docs/label/2023/217242s000lbl.pdf.

[6] Li H., Zuo J., Tang W. (2018). Phosphodiesterase-4 inhibitors for the treatment of inflammatory diseases. Front Pharmacol.

[7] Dong C., Virtucio C., Zemska O. (2016). Treatment of skin inflammation with benzoxaborole phosphodiesterase inhibitors: selectivity, cellular activity, and effect on cytokines associated with skin inflammation and skin architecture changes. J Pharmacol Exp Ther.

[8] Stein Gold L., Alonso-Llamazares J., Draelos Z.D. (2023). Effect of roflumilast cream (ARQ-151) on itch and itch-related sleep loss in adults with chronic plaque psoriasis: patient-reported itch outcomes of a phase 2b trial. Am J Clin Dermatol.

[9] Blauvelt A., Draelos Z.D., Stein Gold L. (2024). Roflumilast foam 0.3% for adolescent and adult patients with seborrheic dermatitis: a randomized, double-blinded, vehicle-controlled, phase 3 trial. J Am Acad Dermatol.

